# Budd-Chiari Syndrome Imaging Diagnosis: State of the Art and Future Perspectives

**DOI:** 10.3390/diagnostics13132256

**Published:** 2023-07-03

**Authors:** Giorgia Porrello, Giuseppe Mamone, Roberto Miraglia

**Affiliations:** 1 Radiology Unit, Department of Diagnostic and Therapeutic Services, IRCCS ISMETT (Mediterranean Institute for Transplantation and Advanced Specialized Therapies), Via Tricomi 5, 90127 Palermo, Italy; gporrello@ismett.edu; 2 Section of Radiology, Department of Biomedicine, Neuroscience and Advanced Diagnostics (Bi.N.D.), Università degli Studi di Palermo, Via del Vespro 127, 90127 Palermo, Italy

**Keywords:** hepatic venous outflow obstruction, prothrombotic state, vein, thrombosis, Budd-Chiari syndrome, ultrasounds, computed tomography, magnetic resonance imaging

## Abstract

Budd-Chiari syndrome (BCS) is a rare hepatic vascular disorder defined by the presence of partial or complete impairment of hepatic venous drainage in the absence of right heart failure or constrictive pericarditis. Several conditions can lead to BCS, from hypercoagulable states to malignancies. Primary BCS is the most common subtype, and usually bartends hypercoagulability states, while secondary BCS involves tumor invasion or extrinsic compression. A combination of clinical and imaging features leads to the diagnosis of BCS, including (1) direct signs: occlusion or compression of the hepatic veins and/or inferior vena cava, and the presence of venous collaterals; (2) indirect signs: morphological hepatic changes with caudate lobe enlargement; inhomogeneous enhancement, and hypervascular nodules. From a clinicopathological point of view, two forms of BCS can be distinguished: acute and subacute/chronic BCS, although asymptomatic and fulminant forms are also possible. Acute presentations are rare, and symptoms include hepatomegaly, ascites, and hepatic insufficiency. Subacute/chronic forms are the most common presentation, with dysmorphic liver and variable degrees of fibrosis deposition. Patients with chronic BCS can develop benign regenerative nodules (large regenerative nodules or FNH [Focal Nodular Hyperplasia]-like lesions), but are also at a higher risk of hepatocellular carcinoma (HCC). The radiologist role is therefore fundamental in both diagnosis and surveillance of BCS. The aim of this review is to present all clinical and imaging signs that can help to reach the diagnosis of BCS, with their clinical significance, providing tips and tricks for the cross-sectional diagnosis of this condition.

## 1. Introduction

Budd-Chiari syndrome (BCS) is a severe vascular liver disease defined by the presence of partial or complete impairment of hepatic venous outflow, in the absence of right heart failure or constrictive pericarditis. BCS most commonly occurs in women between 19 and 49 years of age [1], and has a low incidence and prevalence in the general population with an estimated incidence of 1 new case per million inhabitants per year according to recent metanalyses [2,3]. BCS is still a largely underdiagnosed condition; a high index of suspicion is needed to reach this diagnosis and should be considered in the differential diagnosis of any case of unexplained portal hypertension, and in all forms of acute and chronic liver disease [4]. The diagnosis of BCS is based on the presence of clinical and laboratory findings, associated with radiological evidence of partial or complete venous drainage impairment. Many conditions can lead to BCS, and the site of the obstruction ranges from the smaller hepatic venules to the cavo-atrial junction or the right atrium [5,6,7]. Notably, only hepatic venous outflow tract obstruction of major veins can be visualized on imaging, while small hepatic veins obstruction (known as small vessel BCS) can only be diagnosed on histology [8]. By contrast, in the sinusoidal obstruction syndrome (SOS), previously called veno-occlusive disease, the outflow obstruction arises at level of the sinusoids and terminal hepatic venules rather than the hepatic veins [6]. This latter represents a distinct entity and will not be described, as it eludes from the purposes of this review. According to the underlying occlusion mechanisms, it is possible to distinguish a primary and a secondary form of BCS. The former is the most common one, and it is associated with hypercoagulable states, which cause vascular thrombosis. Another rare cause of primary BCS is membranous web-like obstruction of the inferior vena cava (IVC) that extends to the hepatic veins; this condition is more frequent in Asia and is probably linked to genetic factors [5,6]. Secondary BCS represents <1% of the total cases [9], and is linked to invasion or extrinsic compression of the hepatic veins and/or the IVC, either by benign (e.g., hydatid cysts, hemangiomas) or malignant conditions [5,6,7,8,9,10].

The presence and severity of the symptoms are related to the number of hepatic veins (HV) involved, to the thrombosis extension, and to the impairment of the hepatic functional reserve, due to the increase in intrahepatic sinusoidal pressure and congestion, resulting in sinusoidal dilatation, liver congestion, and hepatomegaly; therefore, a single vein obstruction is often asymptomatic or paucisymptomatic. On this background, portal hypertension builds, leading to the formation of intrahepatic and extrahepatic venous collaterals [5,6,7,8]. If left untreated, BCS progresses to liver failure from fibrosis and eventual death. Nowadays, most patients are treated by endovascular interventions and systemic anticoagulation therapy, which have greatly improved survival [4,10]. Around 10–20% of patients will be referred to liver transplantation (LT), usually due to failure of all other treatments or in case of acute liver failure [11,12]. Endovascular intervention is aimed at relieving the venous outflow obstruction, and techniques include angioplasty, endovascular shunts, and thrombolysis [13]. Trans-jugular portosystemic shunt (TIPS) has an established role, both as a bridge to LT and as a treatment for patients nonresponding to pharmacological therapy or with contraindications to LT [13,14]. This review will provide an overview of the role of imaging in the diagnosis of BCS, presenting the latest improvements and the elements needed to make the diagnosis.

## 2. Diagnosis of Budd-Chari Syndrome

Clinicopathologically, two separated forms of BCS are distinguishable: acute (onset of hepatic outflow obstruction, rarely fulminant) and subacute/chronic (sequelae of remote hepatic outflow obstruction) [1,10]. Most patients will present with chronic forms [3,4,5,6]. The spectrum of clinical presentation varies accordingly, ranging from asymptomatic illness to fulminant liver failure [8,15,16], and is also linked to the site of obstruction and portions of the liver involved. Population-based studies demonstrated that ascites is the most common symptom, occurring in 62–85% of patients, followed by hepatomegaly (67%) [17], pain (61%) [17,18,19], esophageal varices (58%), and gastrointestinal bleeding in 5–21% cases [19]. Around 10–15% of patients will be asymptomatic, presenting only abnormal liver function tests [19]. Concomitant portal vein thrombosis (PVT) is noted in around 10–15% of all BCS patients [20,21] and should therefore always be investigated as it changes the course of the disease [15,16,17]. Indeed, prognosis tends to be worse in BCS-PVT patients [21]. Fulminant BCS, instead, is characterized by acute liver failure, it is determined by a simultaneous acute occlusion of all three HV, and is linked to a high in-hospital mortality [16]. In the acute phase of BCS, affected segments are mostly located at the site of congestion and necrosis, and are hypertrophied. Within a few weeks, the deposition of fibrosis begins, and collaterals start to develop. In the chronic phase, fibrosis will be the dominating aspect, with consequent alteration of liver morphology, atrophy of the affected segments, and hypertrophy of the spared ones [4,5,6,7,8,9,10,11]. Notably, sinusoidal congestion does not develop in the caudate lobe since it presents its own venous drainage, therefore caudate lobe enlargement is seen in around 50% of patients [22,23]. However, this sign is not specific for Budd-Chiari syndrome, as it may be seen in cirrhosis and other chronic liver diseases, in association with splenomegaly and signs of portal hypertension [24]. In later stages, benign regenerative nodules superimpose on the chronic liver background [6,7,25]. The growing amount of fibrosis leads to portal hypertension with all its classical manifestations: ascites, esophageal varices, dilated superficial veins in trunk and varicosities in the lower limbs, intra- or extra-hepatic collaterals, and splenomegaly [4,5,6,7,8,9,10,11,12,13,14,15,16,17,18,19,20,21]. Laboratory findings are not specific. Due to hepatocyte congestion and necrosis, alteration of hepatic function is seen. Extravascular leakage of protein-rich fluids will lead to ascites with high protein content [6,23]. Two peculiar scenarios are acute on chronic BCS and pediatric presentations. The former is the superimposition of a new thrombus on the underlying chronic obstruction, seen in around 15% of cases, which leads to acute presentations and worsened symptoms [10,15,23]. Pediatric patients present with hepatomegaly without ascites and have usually a more silent course, thanks to a more efficient decompression of the portal system, since the presence of potent angiogenic factors leads to rapid collaterals formation [7,8]. In the end, BCS should be kept in mind in cases of newly found ascites with increased liver volume and upper abdominal pain, abundant high-protein ascites, liver disease in patients with thrombogenic conditions, fulminant hepatic failure with increased liver volume, or unexplained chronic liver disease [6,8,15,16]. However, to confirm the clinical suspicion, radiological exams must be performed [15,16,17]. Table 1 resumes all clinical presentations in BCS patients.

## 3. Imaging of Budd-Chiari Syndrome

Imaging diagnosis of BCS is reached with unequivocal radiological confirmation of hepatic venous outflow obstruction [17]. Historically, invasive exams such as hepatic venography and cavography were considered the ‘gold standard’ to diagnose BCS, until the advent of Color-Doppler Ultrasonography (CDUS), which, together with cross-sectional Computed Tomography (CT) and Magnetic Resonance Imaging (MRI), made the non-invasive diagnosis possible. Nowadays, whenever BCS is clinically suspected, guidelines recommend CDUS as the first examination, followed by contrast enhanced CT or MRI to confirm the diagnosis or whenever the imaging and clinic examination are discordant. Venography is now reserved for endovascular treatment of BCS and pre-surgical planning [8,15,17]. CDUS is useful to depict the vascular flux and to check for intra-hepatic thrombosis, while contrast-enhanced CT or MRI scans should be used to confirm the diagnosis of BCS, assess the extent of thrombosis and the degree of the disease, plan radiological intervention, characterize hepatic nodules, exclude malignancies, and evaluate liver morphology changes [4,5,6,7,8,9,10,11,17,18,19,23]. Numerous imaging features have been described in BCS and their presence is related to the stage of the disease, however, reaching the diagnosis is not easy. Liver biopsy is still needed in inconclusive or discordant cases, or in case small hepatic veins involvement should be assessed [6].

In BCS, direct and indirect sign can be distinguished on imaging, in particular:

1. Direct signs: venous anomalies and the visualization of occluded, stagnant, or inverted venous flow in the HV or in the ICV.
2. Indirect signs: non-specific. They represent the consequences of long-standing hepatic venous impairment, including liver parenchymal changes with fibrosis and atrophy of involved segments and hypertrophy of unaffected territories (e.g., caudate lobe hypertrophy). On CT and MRI, centrilobular or sinusoidal congestion, represented as heterogeneous ”mosaic” enhancement after contrast media is also characteristic. Other signs include ascites, portal hypertension, and the presence of benign regenerative nodules, as well as hepatocellular carcinoma [6].

Table 2 sums all imaging findings in relationship to the technique used.

### 3.1. Ultrasounds: What to Look For

CDUS is an excellent first-line imaging modality in BCS [17,26]. According to a recent metanalysis, pooled sensitivities and specificities of CDUS in the diagnosis of BCS are 89% and 68%, respectively [26]. The advantages of CDUS are easy availability, low cost, lack of ionizing radiations, multi-planar imaging, and the ability to provide functional information regarding vascular flow, stenosis, or thrombosis. However, the subjective nature of US, possible technical difficulties, and the restricted evaluation of the extrahepatic collateral pathways pose significant limitations [26]. CDUS obstruction of HV or ICV is the main specific feature to search (80% of patients) [23,24,25,26,27]. CDUS can also give information on the presence of intrahepatic venous collaterals, stenosis, blood flow direction and flow pattern, morphological liver changes, and portal hypertension signs. Frequently encountered non-specific signs are splenomegaly (78%), inhomogeneous liver parenchyma (76%), intrahepatic collaterals (73%), caudate lobe hypertrophy (67%) which may cause ICV compression, ascites (56%), and extrahepatic collaterals (44%) [27]. Another little-known feature to pay attention to is visible and enlarged caudate vein (>3 mm) [8,28]. Among these signs, seeing on US a direct sign plus caudate lobe hypertrophy offers the highest predictive value to identify patients with BCS, carrying a specificity of 100% [15,27,28]. In subacute phase, comma-shaped porto-systemic and intrahepatic collaterals start to develop and are well seen in chronic phase [8]. HV can have a demodulated spectrum (as also observed in patients with steatosis, cirrhosis, or active chronic hepatitis [29]. Finding focal acceleration of blood velocities at the level of stenosis further confirms the presence of venous obstruction. Portal vein, instead, can show slow, hepato-fugal flow [28]. Portal vein thrombosis should be always assessed, especially in known prothrombotic states or portal hypertension, as extension to the superior spleno-mesenteric confluence may contraindicate liver transplantation and poses a risk of intestinal ischemia [6].

### 3.2. Computed Tomography and Magnetic Resonance: What to Look For

CT is used to confirm the diagnosis. It allows a comprehensive evaluation of the hepatic veins, IVC, and intra- and extrahepatic collateral pathways, and helps delineate vascular anatomy prior to TIPS insertion [8]. In a large metanalysis by Gupta et al., pooled sensitivities and specificities of CT in BCS diagnosis are 89% and 72%, respectively [26]. However, CT is less accurate than US and MRI in detecting venous abnormalities, and MRI is considered the best alternative imaging modality for diagnosing BCS [17].

MRI can also better delineate liver parenchymal changes secondary to HV/IVC occlusion and is the best modality to characterize nodules associated with BCS [4,8,26]. MRI also demonstrated the highest sensitivities and good specificities, reaching values of 93% and 55%, respectively, in BCS diagnosis [26]. MRI advantages consist in the possibility of evaluating all the aspects of BCS, with or without contrast medium administration, and without radiation exposure [6,7]. Moreover, it is the only technique that allows the use of hepatospecific contrast media. In acute Budd-Chiari syndrome, occlusion of the hepatic veins with severe ascites is the typical finding [28]. On CT, this will be seen as filling defects of the HV and/or ICV, while on MRI hyperintensity on both T1- and T2-weighted (w) sequences should be assessed. In both, modalities ascites and splenomegaly should always be searched [30].

**Figure 1:**
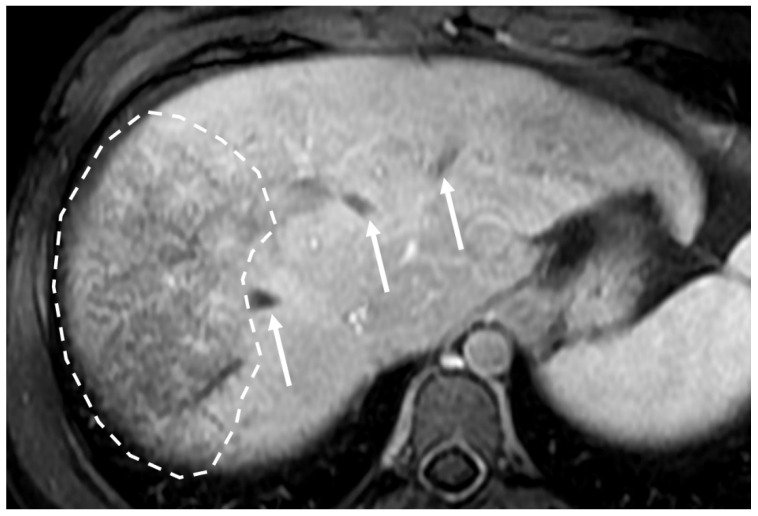
Magnetic resonance (MR) contrast-enhanced portal venous phase shows occlusion of the hepatic veins (arrows) as linear signal hypointensity. Notice the presence of mosaic enhancement (white circle) in the right hepatic lobe.

Chronic HV thrombosis can present as hypoattenuating cords with a narrow diameter on CT [6,7]. Failure to visualize the hepatic veins on CT does not confirm Budd-Chiari syndrome, because they may be difficult to identify on CT, especially in the setting of other conditions, such as advanced cirrhosis. Post-processing tools are important, and HV visualization is better on thick section reconstructed images (MIP, maximum intensity projection) [7]. T2-w sequences can also help to diagnose HV or ICV thrombosis even without contrast media use, thanks to steady-state free-precession (SSFP) or gradient echo sequences [7,31]. T2*-w gradient-recalled echo sequences are particularly useful to demonstrate thrombosis as signal flow void in the hepatic veins and inferior vena cava, which represents a major diagnostic finding [8,25,28]. If contrast media are used, T1-w post-contrast sequences will clearly show the presence of thrombosis and depict its extension (Figure 1). In subacute or chronic BCS, the occluded hepatic veins show linear signal hypointensity on all sequences, and intrahepatic (comma-shaped) collateral vessels are often demonstrated [7,31].

**Figure 2:**
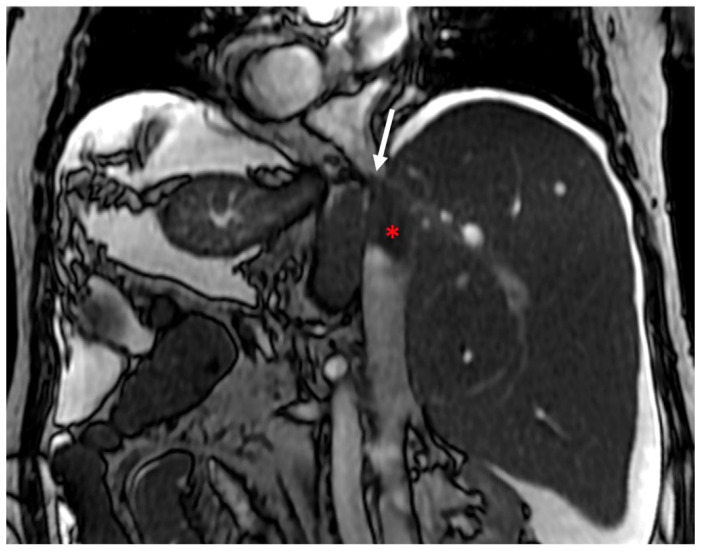
Coronal steady-state free-precession (SSFP) MR sequence exhibits a rare case of primary BCS due to membranous obstruction of the inferior vena cava (arrow) associated with thrombosis in the section below (red asterisk).

**Figure 3:**
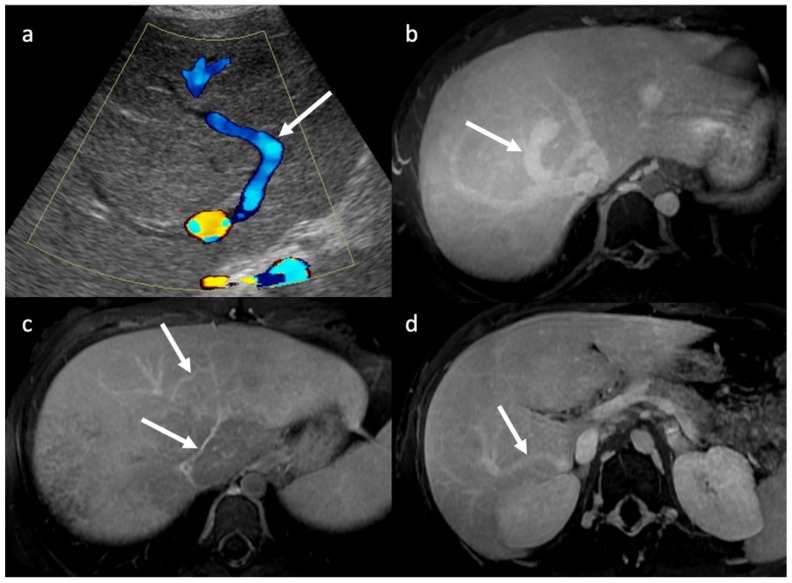
Color-doppler ultrasound (**a**) and axial 3 min delayed phase MR images (**b**–**d**) showing multiple veno-venous shunts (arrows) in different patients, as consequence of HV obstruction.

CT and MR imaging can help to identify the rare cases of primary BCS due to membranous web-like obstruction of the inferior vena cava (IVC) that extends to the hepatic veins (Figure 2). Intrahepatic collateral veins (Figure 3) are the consequence of HV obstruction and are considered an important finding due to their systematic presence in this disease. Some authors have classified these intrahepatic venous collaterals into 4 types: large collaterals draining into the IVC, subcapsular veins, collateral cobwebs, and veno-venous shunts [6,29]. 

In acute BCS, the liver is usually normal or enlarged, and exhibits patchy, decreased peripheral enhancement caused by portal and sinusoidal stasis, with stronger central liver enhancement [28,29,30], a pattern called “flip-flop” [15], or “zonal” enhancement on CT imaging [7].

**Figure 4:**
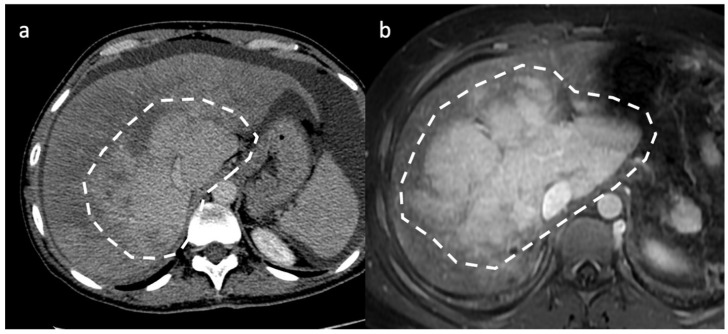
Two different cases of acute/subacute BCS. On axial portal venous phase CT (**a**) and MR (**b**) images the liver exhibits decreased peripheral enhancement caused by portal and sinusoidal stasis, with stronger central liver enhancement, a pattern called “zonal enhancement” (white circles).

T1-w imaging will reveal decreased signal intensity within the liver periphery, with preservation of higher signal intensity within the caudate lobe; T2-weighted sequences instead show heterogeneously increased signal intensity in the peripheral portion of the liver, while the caudate lobe shows normal, hypointense signal intensity [32]. Similarly to CT, in acute and subacute phase, this is mirrored by the “flip-flop” or “zonal” enhancement on dynamic post-gadolinium imaging followed by the typical mosaic aspect on chronic stages (Figure 4) [32,33]. 

**Figure 5:**
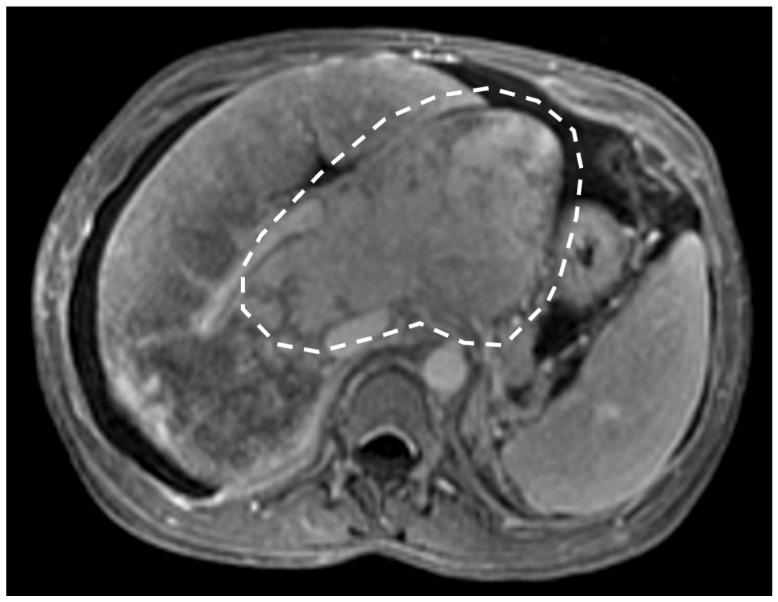
Axial portal venous phase MR image shows huge enlargement and hyperperfusion of caudate lobe (white circle). This is an important finding because sinusoidal congestion does not develop in the caudate lobe since it presents its own venous drainage.

Sinusoidal congestion does not develop in the caudate lobe since it presents its own venous drainage, therefore caudate enlargement will be seen in around 50% of patients and represents an important point of analysis (Figure 5) [22]. Usually it demonstrates compensatory hyperperfusion [8] and when significantly enlarged, it can cause ICV compression. In subacute or chronic Budd-Chiari syndrome, morphological changes and fibrosis occur, following the type of venous involvement.

**Figure 6:**
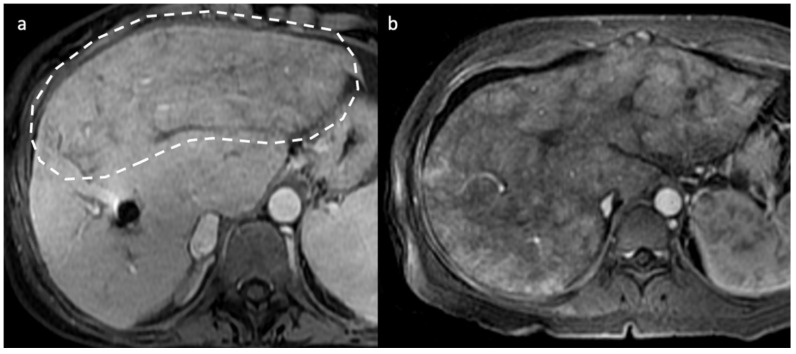
In subacute or chronic BCS morphological changes and fibrosis occur in the liver and regions of hypoperfused parenchyma can still be seen with the so-called ‘‘mosaic’’ enhancement pattern on both CT and MR as a sign of sinusoidal dilatation. Axial portal venous phase (**a**) and arterial phase (**b**) MR images show two different cases of partial (white circle) and diffuse mosaic pattern in the liver, characterized by reticular enhancement.

Regions of hypoperfused parenchyma can still be seen [8,28], with the so-called ‘‘mosaic’’ enhancement pattern (Figure 6) on both CT and MR [33], characterized by homogeneous parenchyma on pre-contrast images, reticular arterial enhancement, and complete or partial homogenization on delayed phase. It is a sign of sinusoidal dilatation and can be transient, typical, but not specific of BCS, as it may be found in all conditions determining sinusoidal alteration (e.g., right cardiac dysfunction, chronic or acute systemic inflammatory states) [6]. 

**Figure 7:**
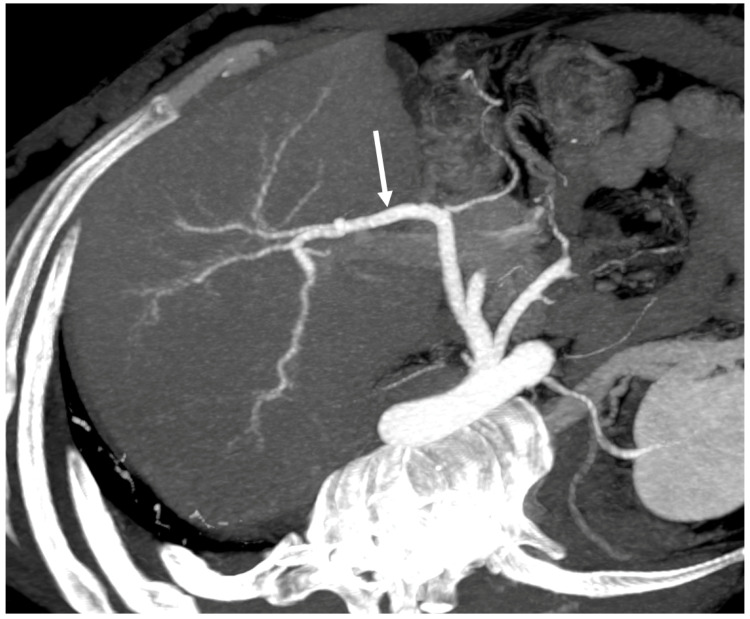
Maximum intensity projection CT reconstruction exhibits the enlargement of the hepatic artery (arrow).

**Figure 8:**
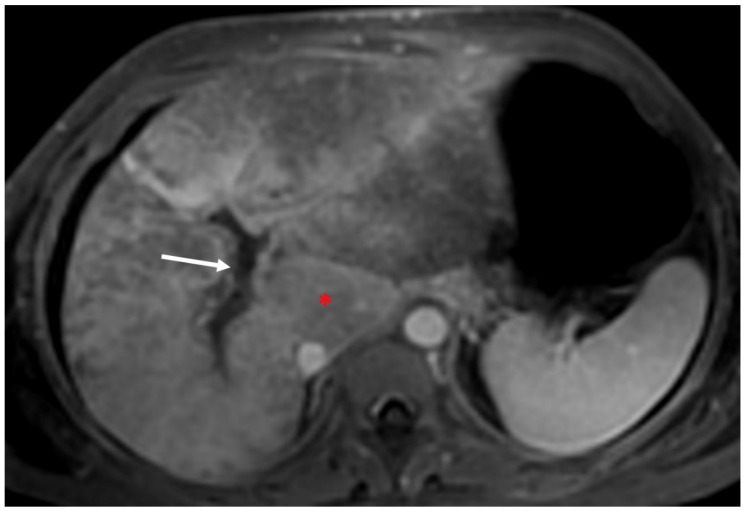
Axial portal venous phase MR image shows the coexistence of portal vein thrombosis (arrow) in patient with BCS. Notice the presence of enlarged caudate lobe (red asterisk).

**Figure 9:**
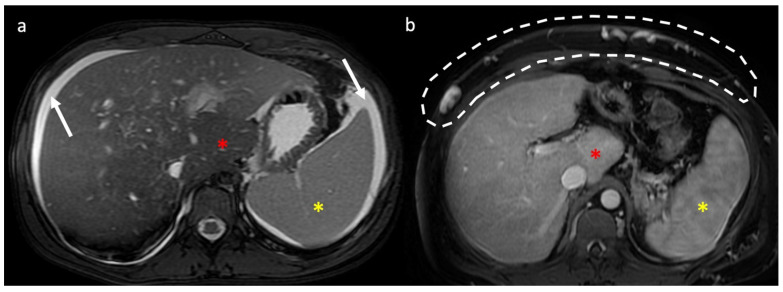
Axial T2 weighted (**a**) and portal venous phase (**b**) MR images show signs of portal hypertension including ascites (arrows), splenomegaly (yellow asterisk), and caput medusae (white circle). Notice the presence of caudate lobe enlargement (red asterisk) in both cases.

In chronic stages, the hepatic artery is usually enlarged compared with the splenic artery (Figure 7) [28], as is the caudate lobe vein (>3 mm) [5,6,7,8,28]. Portal vein thrombosis can develop in chronic BCS as the result of underlying thrombophilia or stagnation of portal flow caused by outflow block (Figure 8) [1,28]. Signs of portal hypertension includes the presence of many different extrahepatic collateral vessels (Figure 9). Cho et al. [34] have proposed a classification of extrahepatic collaterals: (1) veins belonging to the systemic circulation, mostly posterior (lumbar ascending vein, vertebral plexus, azygos, and hemiazygos veins); (2) renal and left hemiazygos veins; (3) phrenic and left pericardiophrenic veins; and (4) abdominal parietal collateral veins (in ICV involvement) when long-standing thrombosis of the ICV shows small calcifications.

Multiphase contrast-enhanced 3D MR angiography (MRA) has recently been increasingly used as an alternative technology for assessing BCS. MRA is an effective, non-invasive method to depict thrombus and level of venous obstruction, and to evaluate the hepatic artery, portal, hepatic veins, and ICV [5,28,35]. MRA can indeed display all the major features of BCS, including vascular thrombosis, stenosis, compressions, and the presence of collaterals [36]. In a large metanalysis, MRA reached a pooled sensitivity of 97.6%, with a specificity of 70.7% [35].

## 4. Hepatic Nodules

**Figure 10:**
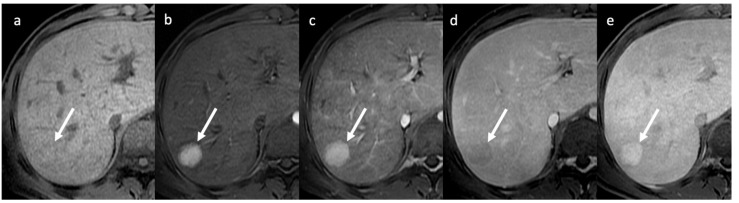
MRI with hepatospecific contrast agent shows a regenerative nodule (arrows) in chronic BCS, isointense on pre-contrast T1 (**a**), markedly and homogeneously hypervascular on arterial and portal venous phases (**b**,**c**), and with wash-out on delayed phase (**d**) mimicking HCC. On hepatobiliary phase (**e**) the lesion appears homogeneously hyperintense to normal liver; this helps to differentiate FNH-like regenerative nodule from HCC.

In the past few decades, the life expectancy of patients with BCS has progressively increased. This brought a higher prevalence of its late complications, including progressive hepatic fibrosis, portal hypertension, hepatic nodules, and an increased risk of hepatocellular carcinoma (HCC) [19,36,37,38,39,40]. There are many hypotheses on the mechanism behind the latter phenomenon; probably it is a consequence of impaired liver venous outflow, associated with portal flow deprivation, and increased hepatic arterialization [25,28,38,39,40,41]. HCC in BCS tends to be even more frequent in case of membranous web-like obstruction of the ICV [7,31,38,39,41], and stricture of hepatic venous outflow tract [40]. Focal liver lesions have a reported prevalence of 36–80% in chronic BCS [17,22,42,43]. Characterization of liver nodules associated with BCS influences the patient’s prognosis [6]. Presence of multiple (>10) nodules, with a typical diameter of 0.5–4 cm, is usually indicative of regenerative nodules (RN) [6,15,25,41], also known as focal nodular hyperplasia-like (FNH-like) nodules, as these two entities share common radiopathological characteristics [7,8,9,10,11,12,13,14,15]. RN originate from vascular disturbances [7,17] and tend to increase in number and size [44], and change their imaging features over time [38]. RN are usually hypo- or hyperechoic, hyperattenuating on pre-contrast CT, markedly and homogeneously hypervascular on arterial phase (on both MR and CT) (Figure 10), and remain slightly hyperattenuating on portal venous and delayed phases [8,25]. MRI characterizes best RN, showing iso-to-hyperintensity on T1-w and predominantly iso-to hypointensity on T2-w images [6,7,8,25]. T1-w hyperintensity could be explained by copper or iron deposits [45] within these nodules or by peri-nodular parenchyma congestion [7]. Rare cases of T2-w hyperintensity have been related to nodules infarction [46,47]. RN do not present restriction and commonly are iso- or hyperintense on hepatobiliary phase. Although benign lesions normally do not have wash-out, many authors have reported the presence of wash-out in regenerative nodules in BCS, thus making the differential diagnosis with HCC harder [7,48]. Classical guidelines for the non-invasive diagnosis of HCC cannot be applied [49] in this context, and pathognomonic pattern has not been univocally detected on CT or MRI [17]. However, on imaging, features favoring HCC in BCS are solitary lesions, usually larger than RN (>30 mm) [15], hypo- or hyperechoic nodules, and hypoattenuating on pre-contrast CT. On MRI, HCC nodules will be hypointense on T1-w, hyperintense on T2-w images, and will show restriction on Diffusion-Weighted Images (DWI) [28]. Contrary to HCC in cirrhotic patients, the arterial enhancement is irregular and heterogeneous [25]. Wash-out on portal venous or delayed phases, heterogeneity, and usually a peripheral capsule on delayed phase images is also reported [25,41], with a predominantly hypointensity on hepatobiliary phase [7]. Tumor invasion of the portal vein was another common finding [50]. The role of Contrat-Enhanced US (CEUS) in differentiating HCC and RN has also been studied, as it better shows the vascularization of the lesion, however MRI remains the most accurate modality.

The prevalence of HCC in patients with Budd-Chiari syndrome varies in the literature, reaching 41% in Japan and 25% in the United States [31]. Other nation-wide series, however, reported percentages as low as 4% [43], and a recent systematic review demonstrated a pooled prevalence of 15.4% [51], suggesting that there are many factors contributing to the development of HCC [41]. Serum alpha fetoprotein (AFP) levels have a role in the differential diagnosis between HCC and regenerative nodules [15,16,17,41,42,43,44,45,46,47,48,49,50,51,52]. As radiological features of hepatocellular nodules arising in the context of vascular liver diseases, including BCS are less characteristic, their diagnosis remains challenging, and may require liver biopsy in the end [7]; however, histologically, HCC nodules in BCS are very-well to well-differentiated hepatocellular proliferations without vascular invasion, leading to a challenging diagnosis with benign proliferations even on pathology [15]. The mean age of development of HCC in patients with BCS varies from 30 to 50 years, although pediatric cases have also been reported [7].

Misdiagnosis of HCC may lead to unnecessary liver transplantation [53]. There is no existing evidence about transformation of RN in HCC, but the number of studies in this field is limited [7]. The only formal recommendation is close and careful multidisciplinary surveillance when suspected nodules are reported [17]. If HCC develops, it should be treated as a routine HCC case by local ablative therapy or LT [15].

### New Techniques, Future Perspectives and Open Questions

Evaluation of portal hypertension, ascitic fluid analysis, upper gastrointestinal endoscopy for variceal assessment, as well as workup for procoagulant state, are an important part of the workup of suspected BCS patients [8]. Endovascular treatment is one of the pillars of the managements of patients with BSC and cross-sectional imaging is important for patient selection and treatment planning. Diagnostic radiologists should make sure to report:

- Number of occluded hepatic veins (when all occluded, angioplasty, and stenting are not indicated)
- Extension of hepatic vein occlusions (short occlusions are better manageable in interventional procedures).
- Visibility and size of the caudate lobe vein
- Presence and size of intrahepatic venous collaterals
- Patency of IVC, intra- and extrahepatic portal vein
- Presence of accessory right hepatic veins [6]

Actual research on BCS is advocating to find more precise way to non-invasively confidently diagnose BCS, to further explore the role of TIPS, and to provide better treatment strategies, especially in paediatric and pregnant population. Future radiological research is oriented towards the development of prognostic scores for the prediction of response and non-response to therapies in individual patients and are searching for more criteria to non-invasively assess the response to treatment, to provide long-term evaluation and, therefore, model the indications of TIPS and HV stenting, especially in younger patients. Recently, numerous papers are also focusing on the emerging role of liver stiffness US measurement in the diagnosis and follow-up of BCS. A study by Dajti et al. [54] demonstrated that high values of both liver (LSM) and spleen (SSM) stiffness are seen in BCS in both acute and chronic presentations, and US elastography could therefore aid the diagnosis of BCS. LSM, indeed, does not solely reflect liver fibrosis but also conditions such as liver congestion, which is one of the mainstays of BCS [54]. SSM is a more recent non-invasive method to assess the degree of portal hypertension, since splenic congestion and related structural changes are direct consequences of increased portal pressure, regardless of its cause. SSM is nowadays considered a direct and more suitable surrogate of portal hypertension than LSM, showing a better performance than LSM in the prediction of clinically significant portal hypertension [55]. However, specific studies in BCS patients are still needed to further evaluate these parameters. Segmental or complete decrease in liver stiffness values after endovascular treatment has also been regarded as a potential marker to assess good response to therapy [37] and to predict BCS compensation over time [55].

## 5. Conclusions

Imaging plays a central role in the diagnosis, treatment, and follow-up of BCS. CDUS and MRI have a particularly important role in the vascular and parenchymal evaluation of the liver. The cornerstone of the diagnosis is the direct identification of venous occlusion of the liver, either at the level of HV or ICV, and the identification of collaterals. BCS is a difficult diagnosis to pose, but should be considered in every case of unexplained liver laboratory alteration, ascites, and hepatomegaly, as well as in any case of acute liver failure.

## Figures and Tables

**Table 1:** Clinical presentations in relationship to each Budd-Chiari Syndrome form.

Budd-Chiari Forms	Clinical Presentation
Acute	Acute hepatic impairment Ascites Pain Kidney impairment and failure
Subacute/Chronic	Impaired liver function Liver morphology alteration (+hypertrophy of caudate) and fibrosis Portal hypertension Ascites Venous collaterals (if IVC obstruction: parietal collaterals) Variceal bleeding Thrombosis
Asymptomatic	Incidentally seen. Multiple chronic venous occlusions Venous collaterals Liver Morphology alteration with fibrosis
Fulminant	Acute liver failure

**Table 2:** Budd-Chiari syndrome findings and peculiar imaging findings related to each technique.

Imaging Technique	Findings
CDUS	HV thrombosis ICV thrombosis and compression (better studied on CT/MRI) PV thrombosis HV demodulation Intra-hepatic collateral vessels Stagnant, reduced and/or hepatofugal PV flow Focal acceleration of velocity corresponding to stenosis Ascites Caudal lobe enlargement
CT	Liver morphology alteration Acute phase: “zonal” or “flip-flop” perfusion Chronic phase: “mosaic” perfusion Extra- and intra-hepatic collaterals Site and extension of thrombosis Pre-endovascular treatment assessment Hepatic artery and caudate vein enlargement Hepatic Nodules Ascites and caudal lobe enlargement
MRI	Characterization of nodules (FNH-like nodules vs. HCC) T1-w decreased signal in hypoperfused regions, corresponding to high T2-w SI T2*-w flow void corresponding to thrombus. Hypointensity along the occluded vessels

List of abbreviations: Color Doppler Ultrasound (CDUS); Computed Tomography (CT); Magnetic Resonance Imaging (MRI); Hepatic Veins (HV); Inferior Cava Vein (ICV); Portal Vein (PV); Signal Intensity (SI); Focal Nodular Hyperplasia (FNH); Hepatocellular Carcinoma (HCC); weighted (w).

## References

[1] Valla D.C. (2018). Budd-Chiari syndrome/hepatic venous outflow tract obstruction. Hepatol. Int..

[2] Li Y., De Stefano V., Li H., Zheng K., Bai Z., Guo X., Qi X. (2019). Epidemiology of Budd-Chiari syndrome: A systematic review and meta-analysis. Clin. Res. Hepatol. Gastroenterol..

[3] Riva N., Ageno W., Qi X. (2020). Epidemiology of Budd-Chiari Syndrome. Budd-Chiari Syndrome.

[4] Bansal V., Gupta P., Sinha S., Dhaka N., Kalra N., Vijayvergiya R., Dutta U., Kochhar R. (2018). Budd-Chiari syndrome: Imaging review. Br. J. Radiol..

[5] Van Wettere M., Bruno O., Rautou P.E., Vilgrain V., Ronot M. (2018). Diagnosis of Budd-Chiari syndrome. Abdom. Radiol..

[6] Mamone G., Carollo V., Di Piazza A., Cortis K., Degiorgio S., Miraglia R. (2019). Budd-Chiari Syndrome and hepatic regenerative nodules: Magnetic resonance findings with emphasis of hepatobiliary phase. Eur. J. Radiol..

[7] Madhusudhan K.S., Sharma S. (2020). Ultrasonography in pediatric Budd-Chiari syndrome. Pediatr. Radiol..

[8] Sharma A., Keshava S.N., Eapen A., Elias E., Eapen C.E. (2021). An Update on the Management of Budd-Chiari Syndrome. Dig. Dis. Sci..

[9] Zhang W., Qi X., Zhang X., Su H., Zhong H., Shi J., Xu K. (2015). Budd-Chiari Syndrome in China: A Systematic Analysis of Epidemiological Features Based on the Chinese Literature Survey. Gastroenterol. Res. Pract..

[10] Langlet P., Escolano S., Valla D., Coste-Zeitoun D., Denie C., Mallet A., Levy V.G., Franco D., Vinel J.P., Belghiti J. (2003). Clinicopathological forms and prognostic index in Budd-Chiari syndrome. J. Hepatol..

[11] Valla D.-C. (2004). Hepatic venous outflow tract obstruction etiopathogenesis: Asia versus the West. J. Gastroenterol. Hepatol..

[12] Aktas H., Ozer A., Yilmaz T.U., Keceoglu S., Can M.G., Emiroglu R. (2022). Liver transplantation for Budd-Chiari syndrome: A challenging but handable procedure. Asian J. Surg..

[13] Giri S., Kale A., Shukla A. (2022). Efficacy and Safety of Transjugular Intrahepatic Portosystemic Shunt Creation for Budd-Chiari Syndrome: A Systematic Review and Meta-Analysis. J. Vasc. Interv. Radiol..

[14] He F., Zhao H., Dai S., Wu Y., Wang L., Huang H., Yue Z., Fan Z., Dong X., Liu F. (2016). Transjugular intrahepatic portosystemic shunt for Budd-Chiari syndrome with diffuse occlusion of hepatic veins. Sci. Rep..

[15] Shukla A., Shreshtha A., Mukund A., Bihari C., Eapen C.E., Han G., Deshmukh H., Cua I.H.Y., Lesmana C.R.A., Al Meshtab M. (2021). Budd-Chiari syndrome: Consensus guidance of the Asian Pacific Association for the study of the liver (APASL). Hepatol. Int..

[16] Thuluvath P.J., Alukal J.J., Zhang T. (2021). Acute liver failure in Budd-Chiari syndrome and a model to predict mortality. Hepatol. Int..

[17] European Association for the Study of the Liver (2016). EASL Clinical Practice Guidelines: Vascular diseases of the liver. J. Hepatol..

[18] Ki M., Choi H.Y., Kim K.A., Kim B.H., Jang E.S., Jeong S.H. (2016). Incidence, prevalence and complications of Budd-Chiari syndrome in South Korea: A nationwide, population-based study. Liver Int..

[19] Darwish Murad S., Plessier A., Hernandez-Guerra M., Fabris F., Eapen C.E., Bahr M.J., Trebicka J., Morard I., Lasser L., Heller J. (2009). EN-Vie (European Network for Vascular Disorders of the Liver). Etiology, management, and outcome of the Budd-Chiari syndrome. Ann. Intern. Med..

[20] Ageno W., Dentali F., Pomero F., Fenoglio L., Squizzato A., Pagani G., Re R., Bonzini M. (2017). Incidence rates and case fatality rates of portal vein thrombosis and Budd-Chiari Syndrome. Thromb. Haemost..

[21] Darwish Murad S., Valla D.C., de Groen P.C., Zeitoun G., Haagsma E.B., Kuipers E.J., Janssen H.L. (2006). Pathogenesis and treatment of Budd-Chiari syndrome combined with portal vein thrombosis. Am. J Gastroenterol..

[22] Vilgrain V., Condat B., Bureau C., Hakimé A., Plessier A., Cazals-Hatem D., Valla D.C. (2006). Atrophy-hypertrophy complex in patients with cavernous transformation of the portal vein: CT evaluation. Radiology.

[23] Leoni F.G., Magnano San Lio P., De Molo C., Bakken S., Ferronato M., Dietrich C.F., Serra C. (2023). Budd-Chiari syndrome (BCS): A challenging diagnosis not to be overlooked-single center report and pictorial essay. J. Ultrasound..

[24] Vilgrain V., Lagadec M., Ronot M. (2016). Pitfalls in Liver Imaging. Radiology.

[25] Brancatelli G., Federle M.P., Grazioli L., Golfieri R., Lencioni R. (2002). Benign regenerative nodules in Budd-Chiari syndrome and other vascular disorders of the liver: Radiologic-pathologic and clinical correlation. Radiographics.

[26] Gupta P., Bansal V., Kumar-M P., Sinha S.K., Samanta J., Mandavdhare H., Sharma V., Dutta U., Kochhar R. (2020). Diagnostic accuracy of Doppler ultrasound, CT and MRI in Budd Chiari syndrome: Systematic review and meta-analysis. Br. J. Radiol..

[27] Boozari B., Bahr M.J., Kubicka S., Klempnauer J., Manns M.P., Gebel M. (2008). Ultrasonography in patients with Budd-Chiari syndrome: Diagnostic signs and prognostic implications. J. Hepatol..

[28] Brancatelli G., Vilgrain V., Federle M.P., Hakime A., Lagalla R., Iannaccone R., Valla D. (2007). Budd-Chiari syndrome: Spectrum of imaging findings. AJR Am. J. Roentgenol..

[29] Bargalló X., Gilabert R., Nicolau C., García-Pagán J.C., Ayuso J.R., Brú C. (2006). Sonography of Budd-Chiari syndrome. AJR Am. J. Roentgenol..

[30] Mathieu D., Vasile N., Menu Y., Van Beers B., Lorphelin J.M., Pringot J. (1987). Budd-Chiari syndrome: Dynamic CT. Radiology.

[31] Dilawari J.B., Bambery P., Chawla Y., Kaur U., Bhusnurmath S.R., Malhotra H.S., Sood G.K., Mitra S.K., Khanna S.K., Walia B.S. (1994). Hepatic outflow obstruction (Budd-Chiari syndrome). Experience with 177 patients and a review of the literature. Medicine.

[32] Noone T.C., Semelka R.C., Siegelman E.S., Balci N.C., Hussain S.M., Kim P.N., Mitchell D.G. (2000). Budd-Chiari syndrome: Spectrum of appearances of acute, subacute, and chronic disease with magnetic resonance imaging. J. Magn. Reason. Imaging.

[33] Mamone G., Miraglia R. (2019). The “mosaic pattern” in hepatic sinusoidal dilatation. Abdom. Radiol..

[34] Cho O.K., Koo J.H., Kim Y.S., Rhim H.C., Koh B.H., Seo H.S. (1996). Collateral pathways in Budd-Chiari syndrome: CT and venographic correlation. AJR Am. J. Roentgenol..

[35] Xu P., Lyu L., Sami M.U., Lu X., Ge H., Rong Y., Hu C., Xu K. (2018). Diagnostic accuracy of magnetic resonance angiography for Budd-Chiari syndrome: A meta-analysis. Exp. Ther. Med..

[36] Lin J., Chen X.H., Zhou K.R., Chen Z.W., Wang J.H., Yan Z.P., Wang P. (2003). Budd-Chiari syndrome: Diagnosis with three-dimensional contrast-enhanced magnetic resonance angiography. World J. Gastroenterol..

[37] Xu P., Lyu L., Ge H., Sami M.U., Liu P., Hu C., Xu K. (2019). Segmental Liver Stiffness Evaluated with Magnetic Resonance Elastography Is Responsive to Endovascular Intervention in Patients with Budd-Chiari Syndrome. Korean J. Radiol..

[38] Panvini N., Dioguardi Burgio M., Sartoris R., Maino C., Van Wettere M., Plessier A., Payancé A., Rautou P.E., Ladouceur M., Vilgrain V. (2022). MR imaging features and long-term evolution of benign focal liver lesions in Budd-Chiari syndrome and Fontan-associated liver disease. Diagn. Interv. Imaging.

[39] Liu F.Y., Wang M.Q., Duan F., Fan Q.S., Song P., Wang Y. (2013). Hepatocellular carcinoma associated with Budd-Chiari syndrome: Imaging features and transcatheter arterial chemoembolization. BMC Gastroenterol..

[40] Li K.S., Guo S., Chen Y.X., Zhang Z.L. (2022). Budd-Chiari syndrome and its associated hepatocellular carcinoma: Clinical risk factors and potential immunotherapeutic benefit analysis. Front. Oncol..

[41] Vilgrain V., Lewin M., Vons C., Denys A., Valla D., Flejou J.F., Belghiti J., Menu Y. (1999). Hepatic nodules in Budd-Chiari syndrome: Imaging features. Radiology.

[42] Moucari R., Rautou P.E., Cazals-Hatem D., Geara A., Bureau C., Consigny Y., Francoz C., Denninger M.H., Vilgrain V., Belghiti J. (2008). Hepatocellular carcinoma in Budd-Chiari syndrome: Characteristics and risk factors. Gut.

[43] Sakr M., Abdelhakam S.M., Dabbous H., Hamed A., Hefny Z., Abdelmoaty W., Shaker M., El-Gharib M., Eldorry A. (2017). Characteristics of hepatocellular carcinoma in Egyptian patients with primary Budd-Chiari syndrome. Liver Int..

[44] Flor N., Zuin M., Brovelli F., Maggioni M., Tentori A., Sardanelli F., Cornalba G.P. (2010). Regenerative nodules in patients with chronic Budd-Chiari syndrome: A longitudinal study using multiphase contrast-enhanced multidetector CT. Eur. J. Radiol..

[45] Wanless I.R. (1990). Micronodular transformation (nodular regenerative hyperplasia) of the liver: A report of 64 cases among 2,500 autopsies and a new classification of benign hepatocellular nodules. Hepatology.

[46] Kim J.A., Ahn H.S., Park H., Seo Y.R., Seo J.H., Kim C.W., Kim H.Y. (2016). Hepatocellular carcinoma hidden by multiple infarcted regenerative nodules. Korean J. Intern. Med..

[47] Kim T., Baron R.L., Nalesnik M.A. (2000). Infarcted regenerative nodules in cirrhosis: CT and MR imaging findings with pathologic correlation. AJR Am. J. Roentgenol..

[48] Khatri G., Merrick L., Miller F.H. (2010). MR imaging of hepatocellular carcinoma. Magn. Reason. Imaging Clin. N. Am..

[49] (2018). American College of Radiology—Liver Reporting & Data System (LI-RADS^®^) Edition. https://www.acr.org/Clinical-Resources/Reporting-and-Data-Systems/LI-RADS/CT-MRI-LI-RADS-v2018.

[50] Takayasu K., Muramatsu Y., Moriyama N., Wakao F., Makuuchi M., Takayama T., Kosuge T., Okazaki N., Yamada R. (1994). Radiological study of idiopathic Budd-Chiari syndrome complicated by hepatocellular carcinoma. A report of four cases. Am. J. Gastroenterol..

[51] Ren W., Qi X., Yang Z., Han G., Fan D. (2013). Prevalence and risk factors of hepatocellular carcinoma in Budd-Chiari syndrome: A systematic review. Eur. J. Gastroenterol. Hepatol..

[52] Van Wettere M., Purcell Y., Bruno O., Payancé A., Plessier A., Rautou P.E., Cazals-Hatem D., Valla D., Vilgrain V., Ronot M. (2019). Low specificity of washout to diagnose hepatocellular carcinoma in nodules showing arterial hyperenhancement in patients with Budd-Chiari syndrome. J. Hepatol..

[53] Oliveira E.C., Duarte A.G., Boin I.F., Almeida J.R., Escanhoela C.A. (2010). Large benign hepatocellular nodules in cirrhosis due to chronic venous outflow obstruction: Diagnostic confusion with hepatocellular carcinoma. Transplant Proc..

[54] Taniguchi T., Ohtani T., Kioka H., Tsukamoto Y., Onishi T., Nakamoto K., Katsimichas T., Sengoku K., Chimura M., Hashimoto H. (2019). Liver Stiffness Reflecting Right-Sided Filling Pressure Can Predict Adverse Outcomes in Patients with Heart Failure. JACC Cardiovasc. Imaging.

[55] Dajti E., Ravaioli F., Colecchia A., Marasco G., Vestito A., Festi D. (2019). Liver and Spleen Stiffness Measurements for Assessment of Portal Hypertension Severity in Patients with Budd Chiari Syndrome. Can. J. Gastroenterol. Hepatol..

